# Integrated bioinformatics and clinical validation reveals PRDX6 as a mitochondrial hub gene in systemic lupus erythematosus

**DOI:** 10.1007/s10067-025-07860-8

**Published:** 2026-01-06

**Authors:** Xingyu Liu, Yan Xiao, Yaxin Deng, Jie Chen, Ting Peng, Yixin Jin, Qian Dai, Mei Zeng

**Affiliations:** 1 Institute of Rheumatology and Immunology, the Affiliated Hospital of North Sichuan Medical College, 1# South Maoyuan Road and Institute of Basic Medicine, North Sichuan Medical College, 234# Fujiang Road, Nanchong, 637001 Sichuan Province China; 2 Medical Imaging Key Laboratory of Sichuan Province, the Affiliated Hospital of North Sichuan Medical College, 1# South Maoyuan Road, Nanchong, Sichuan 637001 China; 3 North Sichuan Medical College Innovation Centre for Science and Technology, North Sichuan Medical College, 234# Fujiang Road, Nanchong, 637001 China

**Keywords:** Mitochondria-related genes, Organ involvement, Prdx6, SLE, SLEDAI score

## Abstract

**Objectives:**

Peroxiredoxin 6 (PRDX6), a potent antioxidant enzyme, has garnered considerable interest for its potential involvement in inflammatory diseases. However, the relationship between PRDX6 and SLE remains poorly understood. This study aims to elucidate the association between PRDX6 and SLE, providing insights into its potential role in disease mechanisms.

**Method:**

Gene expression datasets (GSE50772, GSE61635) from the GEO database were merged and batch-corrected (sva, limma). DEGs were identified and SLE-associated gene modules were analyzed by weighted gene co-expression network analysis (WGCNA). Intersecting differentially expressed genes (DEGs), module genes, and MitoCarta3.0-defined mitochondria-associated DEGs, which were refined by LASSO, Random Forest, and SVM-RFE to select hub genes. The expression levels of PRDX6 in peripheral blood mononuclear cells (PBMCs) from SLE patients were measured by quantitative PCR and Western blot analysis.

**Results:**

A total of 1581 DEGs (865 upregulated, 716 downregulated) were identified. WGCNA revealed a key module of 1615 genes; intersecting this module with DEGs and MitoCarta3.0 yielded 36 mitochondria-associated DEGs. Three machine learning methods narrowed these to 11 core genes, including PRDX6. The expression levels of PRDX6 were significantly lower in SLE PBMCs than in healthy controls, especially in active SLE. Patients with renal or joint involvement had lower PRDX6 levels. The mRNA levels of PRDX6 showed an inverse correlation with Systemic Lupus Erythematosus Disease Activity Index (SLEDAI) score.

**Conclusions:**

This study suggested that PRDX6 might be a potential biomarker and exert protective effects by reducing oxidative stress in SLE.

**Table:** 

**Key Points** • *IFI27 and PRDX6 serve as potential mitochondrial-related biomarkers in SLE.* • *PRDX6 is significantly downregulated in SLE.* • *PRDX6 is associated with SLEDAI score.*

## Introduction

SLE is a chronic autoimmune disease with a genetic predisposition, characterized by excessive autoantibody production and impaired immune complex clearance, leading to sustained inflammation and multi-organ damage [1]. The pathogenesis involves a complex interplay of genetic predisposition, environmental triggers, hormonal influences, and immune dysregulation, with the exact mechanistic pathways yet to be fully elucidated [2]. The diagnosis of SLE is currently based on two widely accepted international classification criteria. Due to its multi-organ manifestations and overlapping features with infections, malignancies, and other systemic diseases, SLE poses significant diagnostic challenges, particularly in the early stages.

Emerging research has established that mitochondrial dysfunction, encompassing both structural alterations and bioenergetic deficits, serves as a key driver of inflammatory cascade initiation and immune cell hyper-activation in SLE pathogenesis [3–5]. Oxidative stress-induced mitochondrial dysfunction generates excessive mitochondrial reactive oxygen species (mtROS) and oxidation of mitochondrial DNA (Ox-mtDNA), which serve as damage-associated molecular patterns (DAMPs) to activate the NLRP3 inflammasome, creating a feed-forward loop that sustains chronic inflammation [6]. Clinically, environmental triggers including ultraviolet radiation and viral infections significantly elevate ROS levels in SLE patients. This oxidative overload exacerbates redox imbalance, promotes immune dysregulation, triggers pathological cell death pathways, and ultimately fuels the generation of pathogenic auto-antibodies [7, 8]. These alterations drive mitochondrial dysfunction and systemic metabolic disruption, mechanistically underpinning multi-organ pathology of SLE. Therefore, mitochondria-targeted therapies, particularly those addressing bioenergetic failure and redox imbalance, are revolutionizing SLE treatment strategies by targeting disease pathogenesis at its root.

Advances in bioinformatics have enabled high-throughput screening of mitochondrial-associated DEGs, providing critical insights into molecular biomarkers of SLE pathogenesis. In the present study, we retrieved two publicly available SLE transcriptomic datasets (GSE50772 and GSE61635) from the GEO database. Following rigorous batch effect correction using ComBat, DEGs were identified through limma-voom pipeline. Subsequently, weighted gene co-expression network analysis (WGCNA) was employed to construct gene modules, with particular emphasis on mitochondrial-associated modules demonstrating highest connectivity. By intersecting DEGs with the MitoCarta3.0 database of annotated mitochondrial genes, we derived a set of mitochondria-associated DEGs. To prioritize the most biologically relevant candidates, we implemented a tri-modal machine learning approach: (1) Least Absolute Shrinkage and Selection Operator (LASSO) regression for feature selection, (2) Support Vector Machine-Recursive Feature Elimination (SVM-RFE) for dimensionality reduction, and (3) Random Forest classifier for importance ranking. This integrative strategy identified 11 high-confidence mitochondrial hub genes, and ROC curve analysis was performed to evaluate the diagnostic performance of these core genes. To investigate the expression patterns of these core genes in SLE patients, we quantified their mRNA levels in PBMCs using quantitative reverse transcription PCR (qRT-PCR). Further statistical analyses examined correlations between PRDX6 expression and SLE disease activity (SLEDAI) as well as organ involvement.

## Methods

### Download and processing of data

The expression profiles of GSE50772 (with 20 normal samples and 61 SLE samples) and GSE61635 (with 30 normal samples and 79 SLE samples) were downloaded from the GEO database to obtain DEGs between SLE and healthy samples. Another independent dataset, namely GSE72509 (with 18 normal samples and 99 SLE samples), was used as a validation set. The microarray data were converted into log2 values, and the combat algorithm implemented in the R package “sva (version 3.40.0)” was employed for integration to remove the batch effect and form a combined dataset.

### Differential gene expression analysis and functional analysis

DEGs were identified by performing differential expression analysis between SLE samples and control samples using the R package “limma (version 3.50.3)” (criteria: |logFC|> 1.5 and adjusted *P* value < 0.05). A volcano plot and heatmap were generated to show significantly upregulated and downregulated genes. The DAVID database was used to analyze the functions of the DEGs based on gene ontology (GO) and Kyoto Encyclopedia of Genes and Genomes (KEGG) pathway.

### Establish a WGCNA co-expression network

The WGCNA package in R was employed to construct a co-expression network aimed at DEGs for identifying potential functional modules of SLE samples. Based on the weighted correlation adjacency matrices and cluster analyses, genes with similar expression patterns were assigned to co-expression modules. The topological overlap matrix (TOM), derived from the adjacency matrix, was used to allocate genes into different modules according to the differences among them in the TOM. Notably, the cut height was set to 140 and the soft-thresholding power was set as 9 (scale-free *R*^2^ = 0.95). Additionally, both gene importance (GS) and module membership (MM) were analyzed. The Spearman correlation coefficients and the corresponding *P* values were also analyzed. Eventually, the corresponding genes extracted from the hub module were obtained for further analysis.

### Validation of the classification model for SLE

To evaluate the classification accuracy of the model, we calculated ROC curves and area under the curve (AUC) in both the training and validation sets, generated with the pROC R package.

### Study subjects and baseline demographic and clinical characteristics

This study enrolled 70 SLE patients (67 females, 3 males; mean age 41.9 ± 14.68 years, range 14–75) from the Department of Rheumatology at the Affiliated Hospital of North Sichuan Medical College between April and December 2024. All patients fulfilled the 2019 EULAR/ACR classification criteria for SLE [36]. Exclusion criteria comprised (1) pregnancy, (2) active infection, (3) malignancy, (4) psychiatric disorders, (5) comorbid autoimmune diseases, and (6) significant chronic illnesses.

Forty-five age- and sex-matched healthy controls (42 females, 3 males; mean age 38.16 ± 16.85 years, range 11–69) were recruited from the hospital’s health examination center during the same period. Demographic analysis confirmed no significant intergroup differences in age (*P* = [exact value]) or sex distribution (*P* = [exact value]) using (specific statistical test, e.g., independent *t*-test and *χ*^2^ test).

Comprehensive laboratory parameters were systematically collected, including: hematological indices (complete blood count), renal function markers (urinalysis, 24-h urinary protein), immunological profiles (complement C3/C4 levels, anti-dsDNA antibody titers), and clinical manifestations (oral ulcers, alopecia, cutaneous rash, arthritis). Disease activity was quantified using the Systemic Lupus Erythematosus Disease Activity Index (SLEDAI), revealing 41 patients (58.6%) with mild/inactive disease (SLEDAI < 10) and 29 patients (41.4%) with moderate-severe activity (SLEDAI ≥ 10). Patients were stratified by organ system involvement: mucocutaneous (56.2% prevalence), hematologic (42.8% prevalence), musculoskeletal (38.7% prevalence), and renal (34.1% prevalence). Complete demographic and clinical characteristics are detailed in Tables 1 and 2, respectively.

**Table 1:** Basic information of the HC and SLE group

	HC group (*n* = 45)	SLE group (*n* = 70)	*t*/*χ*^2^	*P* value
Age (*x* ± *s*)/year	38.16 ± 16.85	41.91 ± 14.68	− 1.264	0.209
Sex (female/male)/case	42/3	66/4	0.000	1.000

**Table 2:** Basic information of SLE patients

Organ involvement	With/case (%)	Without/case (%)
Kidney	28 (40)	42 (60)
Joint	13 (18.6)	57 (81.4)
Mucocutaneous lesions	27 (38.6)	43 (61.4)
Hematologic manifestations	39 (52.9)	31 (47.1)

#### RT‑qPCR analysis of IFI27 and PRDX6 mRNA levels in PBMCs

Total RNA was extracted from PBMCs using TRIzol reagent (Invitrogen, USA) and quantified by spectrophotometry (A₂₆₀/A₂₈₀ 1.8–2.0). First‑strand cDNA was synthesized from 500 ng–1 µg of RNA using the HiScript III RT SuperMix (Vazyme, China). Quantitative PCR was performed on a LightCycler 480 (Roche, Switzerland) with SYBR Green Master Mix (Takara, Japan) under the following conditions: 95 °C for 30 s, then 40 cycles of 95 °C for 10 s and 60 °C for 30 s, followed by melt‑curve analysis. PCR amplification was performed using the following primers: PRDX6 (forward, 5′-AGCTGTCTATCCTCTACCCAG-3′; reverse, 5′-GACCATCACACTATCCCCATC-3′), IFI27 (forward, 5′-CCATTCTAGCTGGTTCGAAGG-3′; reverse, 5′-CAAGGTGGAGGCAGAGAAC-3′), and β‑actin (forward, 5′-GAGCGGGAAATCGTGCGT-3′; reverse, 5′-TCATTGCCAATGGTGATGACCT-3′).

### Correlation of PRDX6 mRNA levels with clinical parameters

Spearman’s rank correlation analysis was performed to evaluate (i) the association between PRDX6 mRNA levels in PBMCs from SLE patients and disease activity (SLEDAI score), (ii) systemic inflammatory markers (ESR, CRP), (iii) immunological parameters (anti-dsDNA antibody titers, complement C3/C4 levels), and (iv) organ-specific involvement (renal, articular, mucocutaneous, and hematologic manifestations).

### Western blot analysis of PRDX6 protein levels in PBMCs

Total protein was extracted from PBMCs using RIPA buffer (Thermo Fisher, USA) and quantified by BCA assay (Pierce, USA). Equal amounts of protein (20 µg) were separated on 10% SDS–PAGE gels and transferred to PVDF membranes (Millipore, USA). Membranes were blocked in 5% non‑fat milk in TBST for 1 h at room temperature, then incubated overnight at 4 °C with anti‑PRDX6 (ab92322, 1:1000) and anti‑β‑actin (1:5000) primary antibodies. After three 10 min washes in TBST, membranes were incubated with HRP‑conjugated secondary antibodies (1:5000) for 1 h at room temperature, followed by four 15 min TBST washes. Bands were detected using an ECL kit (Thermo Fisher, USA) and quantified with ImageJ software.

### Immune cell composition

To analyze the immune cell composition, we employed the CIBERSORT algorithm to compute the proportions of different immune cell types in peripheral blood mononuclear cells (PBMCs) from both SLE patients and healthy individuals from GEO databases, using the gene expression matrix as the basis. For visualization, the R package “vioplot” was applied to generate plots showing the distribution of 22 immune cell proportions in the SLE group versus the control group. Meanwhile, the “corrplot” package was used to construct a heatmap, which illustrates the quantitative correlation patterns among various immune cell populations. Furthermore, to explore potential links between diagnostic marker expression levels and immune cell ratios, we utilized the “ggplot2” R package for related analyses and visualizations.

### Statistical analysis

Data were entered and processed in SPSS 27.0. Bar graphs were generated with GraphPad Prism 10, and all other plots in R 4.4.1. Continuous variables were tested for normality by the Kolmogorov–Smirnov test: normally distributed data are presented as mean ± SD and compared by an unpaired *t*‑test; non‑normal data are presented as median (interquartile range) and compared by the Mann–Whitney *U* test. Categorical variables are expressed as percentages and analyzed by the *χ*^2^ test. Correlations between two variables were assessed by Pearson’s or Spearman’s correlation analysis, depending on distribution. Two‑tailed *P* < 0.05 was considered statistically significant.

## Results

### Identification of DEGs in SLE

**Fig. 1:**
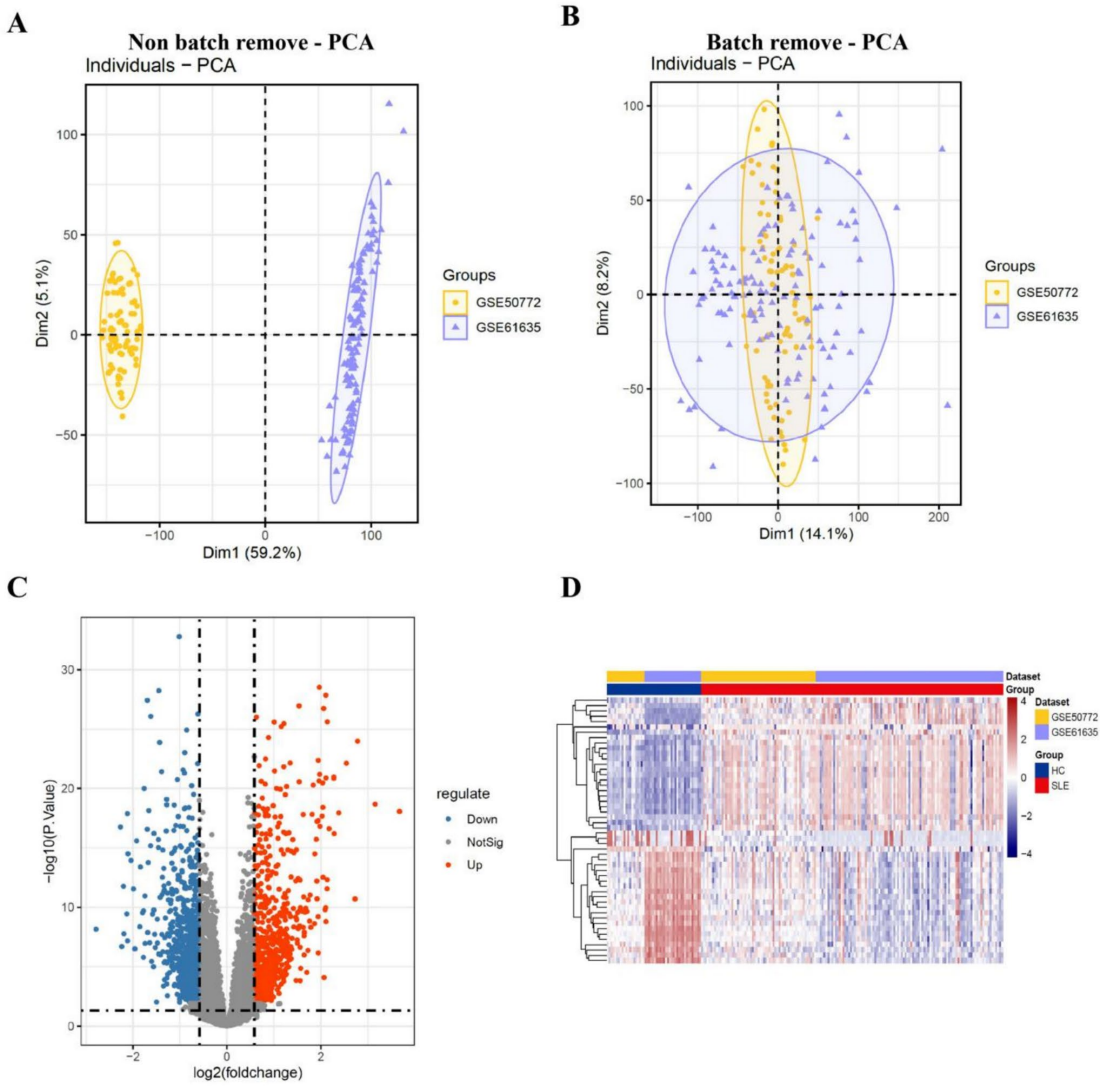
Identification of differentially expressed genes (DEGs). (**A**, **B**) Principal component analysis (PCA) plots of the combined gene expression profiles before and after batch-effect correction with ComBat, respectively. (**C**) Volcano plot visualizing the identified DEGs, using thresholds of |fold change| 1.5 and adjusted P 0.05. (**D**) Heatmap showing the expression patterns of DEGs across samples from systemic lupus erythematosus (SLE) patients and healthy controls

Gene expression profiles were retrieved from the Gene Expression Omnibus (GEO) database (accession numbers: GSE50772 and GSE61635). These datasets comprised transcriptomic data from PBMCs, including GSE50772 (61 systemic lupus erythematosus (SLE) patients and 20 healthy controls) and GSE61635 (99 SLE patients and 30 healthy controls). Principal component analysis (PCA) was employed to identify inherent batch differences in the training set (Fig. 1A). To improve the efficacy of the subsequent analysis, we used the “ComBat” algorithm to address the batch effects. The batch-correction methods successfully eliminated the batch effects to a certain degree (Fig. 1B). Then, differential expression analysis was performed on the training set to screen for DEGs. A total of 1581 significant DEGs associated with SLE were identified, including 865 upregulated and 716 downregulated genes. The results were shown in a volcano plot (Fig. 1C). In addition, the top 25 upregulated and top 25 downregulated genes were displayed in a heatmap (Fig. 1D).

### Functional annotation and pathway enrichment of DEGs

**Fig. 2:**
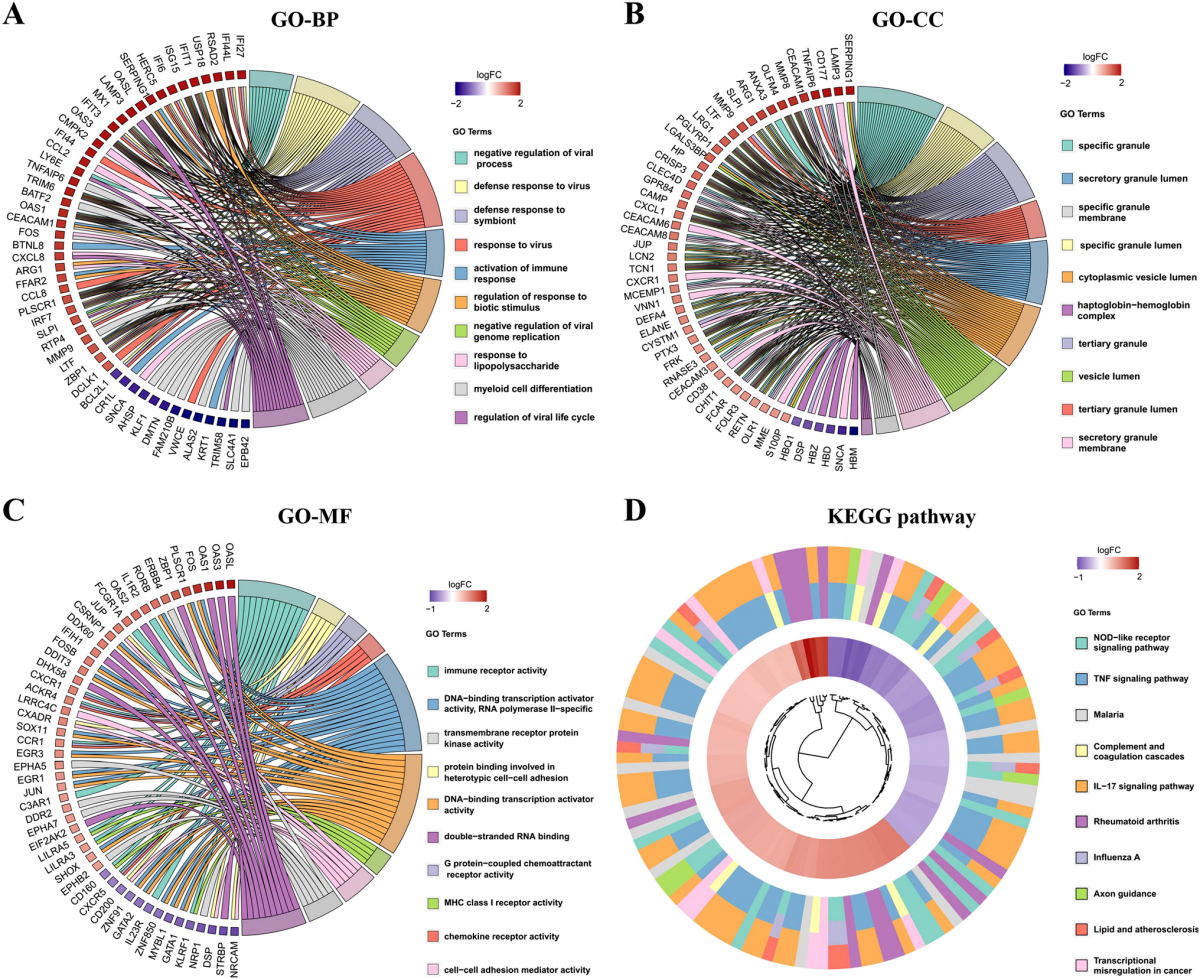
Functional annotation and pathway enrichment of DEGs. **A** Top 10 biological process (BP) GO pathways. **B** Top 10 cellular component (CC) GO pathways. **C** Top 10 molecular function (MF) GO pathways. **D** Top 10 KEGG pathways. DEGs, differentially expressed genes; GO, gene ontology; KEGG, Kyoto Encyclopedia of Genes and Genomes

Based on Gene Ontology Biological Process (GO-BP) analysis, the DEGs were significantly enriched in pathways related to immune response activation, including negative regulation of viral processes, defense response to viruses and symbionts, regulation of biotic stimulus responses, inhibition of viral genome replication, cellular response to lipopolysaccharide, myeloid cell differentiation, and modulation of viral life cycle (Fig. 2A). This pattern of enrichment suggests a robust activation of antiviral and innate immune mechanisms in the studied biological context. GO Cellular Component (GO-CC) analysis revealed significant enrichment of DEGs in granule-related compartments and secretory lumens of immune cells (Fig. 2B), suggesting active involvement in vesicular trafficking and immune effector functions. Gene Ontology Molecular Function (GO-MF) enrichment analysis highlighted significant involvement of DEGs in key functional categories, including immune receptor activity, heterotypic cell–cell adhesion (via protein binding), G protein-coupled chemoattractant receptor activity, chemokine receptor activity, RNA polymerase II-specific transcription activation (DNA-binding), MHC class I receptor activity, cell–cell adhesion mediation, transmembrane receptor protein kinase activity, and double-stranded RNA binding (Fig. 2C). These findings suggest coordinated regulation of immune recognition, cell signaling, and transcriptional control mechanisms. KEGG pathway analysis further demonstrated that the DEGs were significantly enriched in several functionally relevant pathways (FDR < 0.05), innate immune activation (NOD-like receptor signaling pathway, complement and coagulation cascades, influenza A infection pathway), inflammatory signaling (TNF signaling pathway, IL-17 signaling pathway), metabolic and vascular regulation (lipid metabolism and atherosclerosis, axon guidance), and disease-associated pathways (transcriptional misregulation in cancer, rheumatoid arthritis pathogenesis, malaria infection response) (Fig. 2D).

### Identification of mitochondrial core genes in SLE via WGCNA and differential expression analysis

**Fig. 3:**
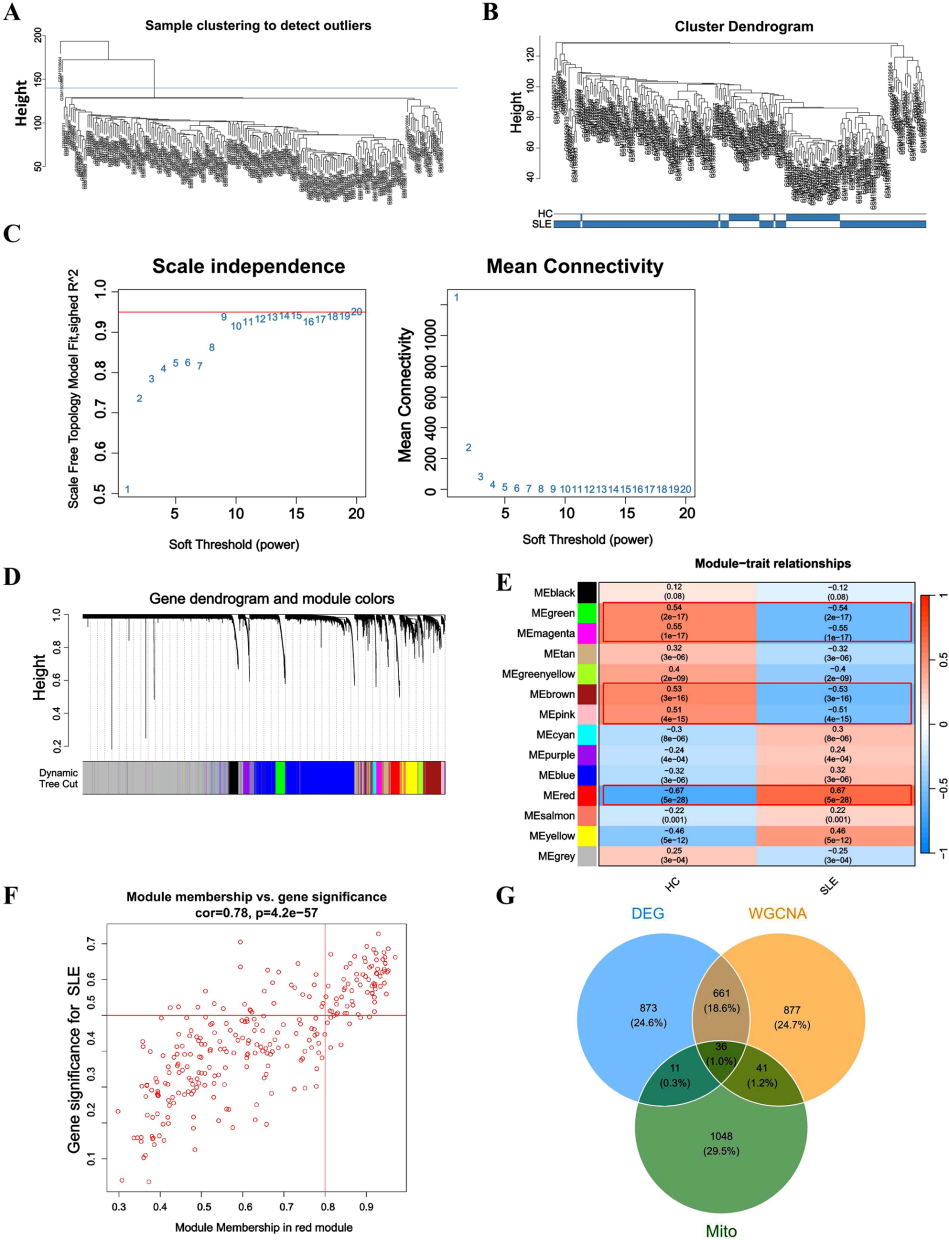
WGCNA. **A**, B Sample clustering of merged dataset to detect outliers. Two outlier samples (GSM1509664, GSM1509669) were excluded, and all remaining samples were selected for further analysis. **C** Determine the best soft threshold. The soft threshold value of 9 was determined as the optimal choice for constructing a scale-free network based on the position of the red line (*R*^2^ = 0.9). **D** Origin and merged modules displaying under the clustering tree for merged dataset. **E** Heatmap of the correlation between module eigengenes and the occurrence of SLE. **F** Scatterplot of correlations between gene significance (GS) and module membership (MM) in red modules. **G** The Venn diagram showing the intersection of the diagnostic biomarkers screened by the DEGs, key module genes, and mitochondria-associated genes

Given the large size of the gene expression matrix (19,746 genes across 210 samples), we performed an initial gene filtering step to reduce dimensionality by retaining the top 50% most variable genes. This resulted in a refined data set of 9873 genes for subsequent WGCNA analysis (Fig. 3A). Potential outlier samples were assessed via hierarchical clustering, leading to the exclusion of two samples (GSM1509664, GSM1509669) due to divergent expression profiles. The final data set comprised 208 samples (50 HC, 158 SLE) (Fig. 3B). According to the WGCNA methodology, a scale-free network was constructed with a soft threshold power of 9 (scale-free topology fit index *R*^2^ = 0.95) (Fig. 3C). Subsequently, we performed Pearson correlation analysis between module eigengenes (MEs) and SLE clinical status. The red module had the strongest positive relation with SLE (*r* = 0.65), while the green, magenta, brown, and pink modules showed strong negative correlations, with Pearson’s *r* values of − 0.82, − 0.79, − 0.85, and − 0.76, respectively (Fig. 3D, E). Based on the above criteria, five modules with |Cor|> 0.5 were selected, comprising a total of 1615 genes. Venn analysis was performed sequentially on (1) 1615 WGCNA module genes and 1581 DEGs (697 overlapping genes), then (2) the resulting gene set with 1151 mitochondria-related genes in SLE, ultimately identifying 36 high-priority mitochondrial core genes (Fig. 3F).

### Multi-algorithm machine learning prioritizes mitochondrial dysfunction-related hub genes for SLE diagnosis

**Fig. 4:**
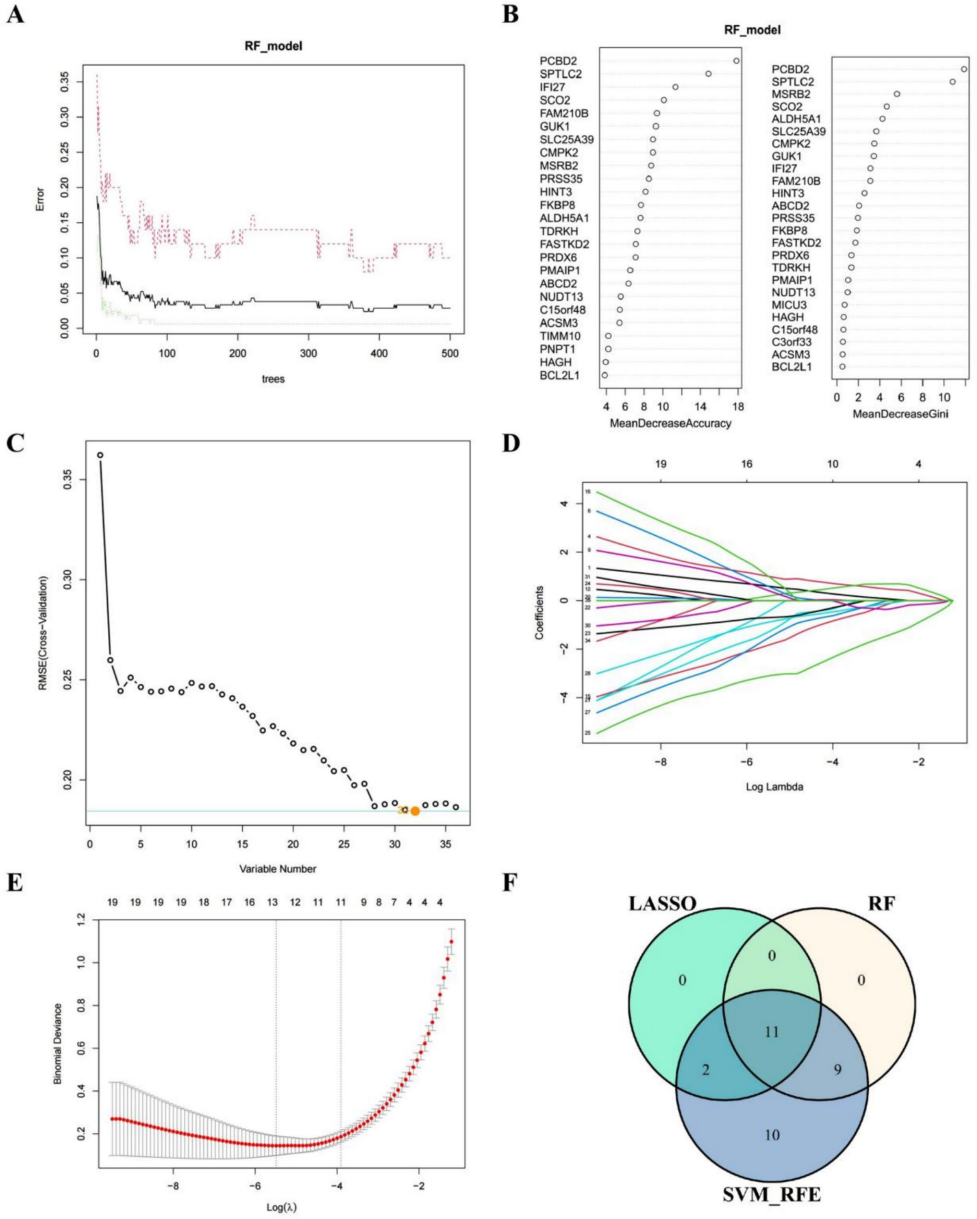
Identification of hub genes for SLE. **A**, **B** Based on RF algorithm to screen biomarkers. **C** SVM-RFE algorithm screening diagnostic biomarkers. **D**, **E** LASSO logistic regression algorithm to screen diagnostic markers. **F** Venn diagram of key characteristic genes; overlapping genes selected as hub genes. SLE, systemic lupus erythematosus; RF, Random Forest; SVM-RFE, Support Vector Machine-Recursive Feature Elimination; LASSO, Least Absolute Shrinkage and Selection Operator

To further screen the hub genes with the most diagnostic values, three machine learning methods—Lasso regression, SVM-RFE (Support Vector Machine-Recursive Feature Elimination), and RF (Random Forest)—were employed to further screen and identify 36 mitochondria-related DEGs. The RF classifier achieved minimal error and maximal stability with 383 trees, indicating an optimal ensemble size (Fig. 4A). The Random Forest analysis identified PCBD2, SPTLC2, and IFI27 as the most influential genes, based on their maximal MeanDecreaseGini scores among the top 20 candidates (Fig. 4B). The SVM-RFE model achieved optimal classification performance with 32 candidate genes, exhibiting the minimal root-mean-square error (Fig. 4C). These 32 genes were consequently identified as key signature genes. At the same time, 13 genes were screened from DEGs by LASSO logistic regression (Fig. 4D, E). Ultimately, IFI27, MSRB2, PMAIP1, SCO2, SPTLC2, ABCD2, FKBP8, PCBD2, PRDX6, PRSS35, and TDRKH were identified as hub genes based on their consistent selection across all three algorithms (LASSO, SVM-RFE, and RF) (Fig. 4F).

### Validation of mitochondrial hub gene expression and diagnostic performance in SLE across independent cohorts

**Fig. 5:**
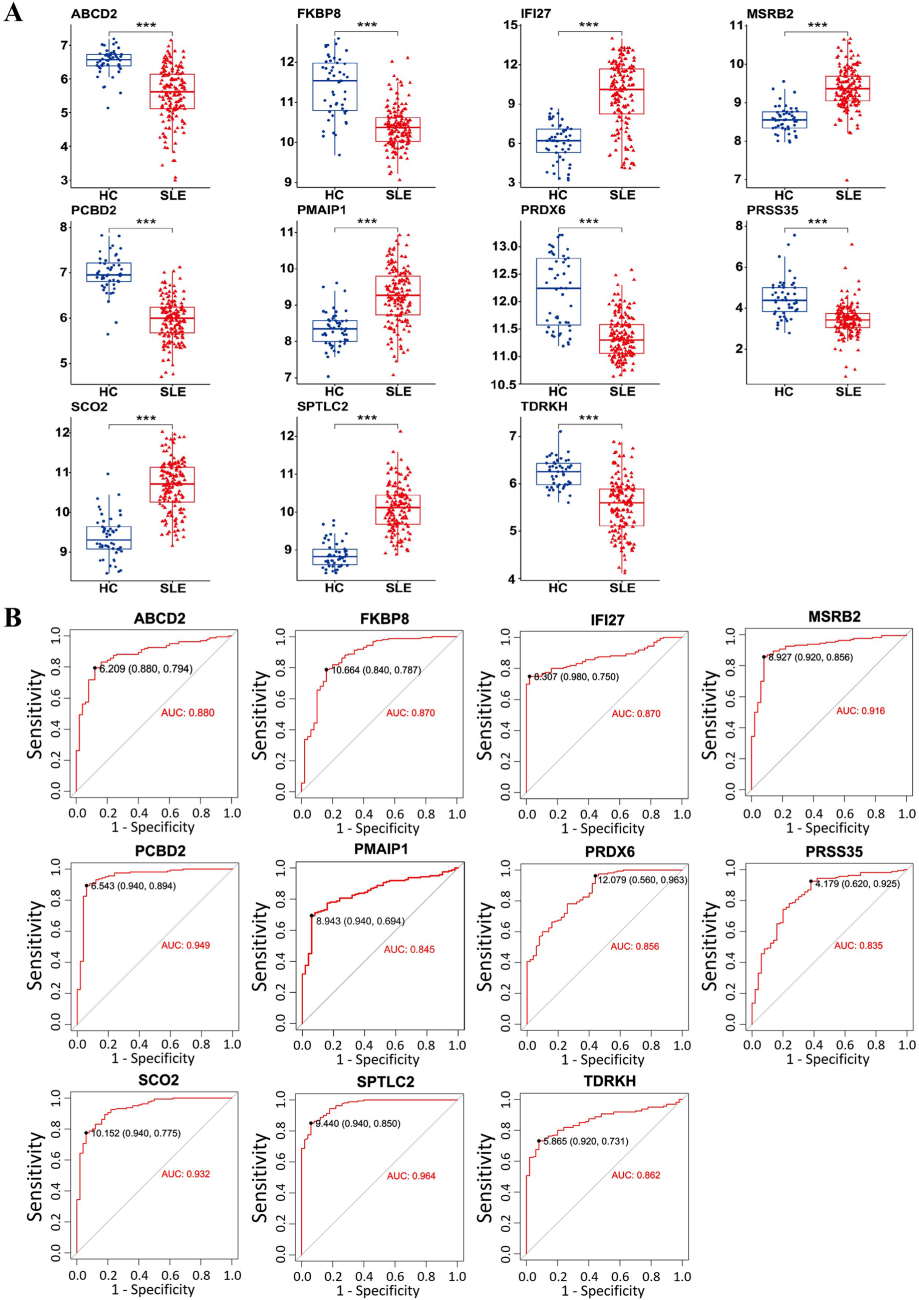
The expression levels of hub genes. **A** Validation of the expression of diagnostic biomarkers based on the training datasets combined by GSE50772 and GSE61635. “HC” represented the health control samples, and “SLE” represented the SLE patients. **B** The ROC curve showing the AUC value of IFI27, MSRB2, PMAIP1, SCO2, SPTLC2, ABCD2, FKBP8, PCBD2, PRDX6, PRSS35, and TDRKH based on the data set of the discovery cohort

**Fig. 6:**
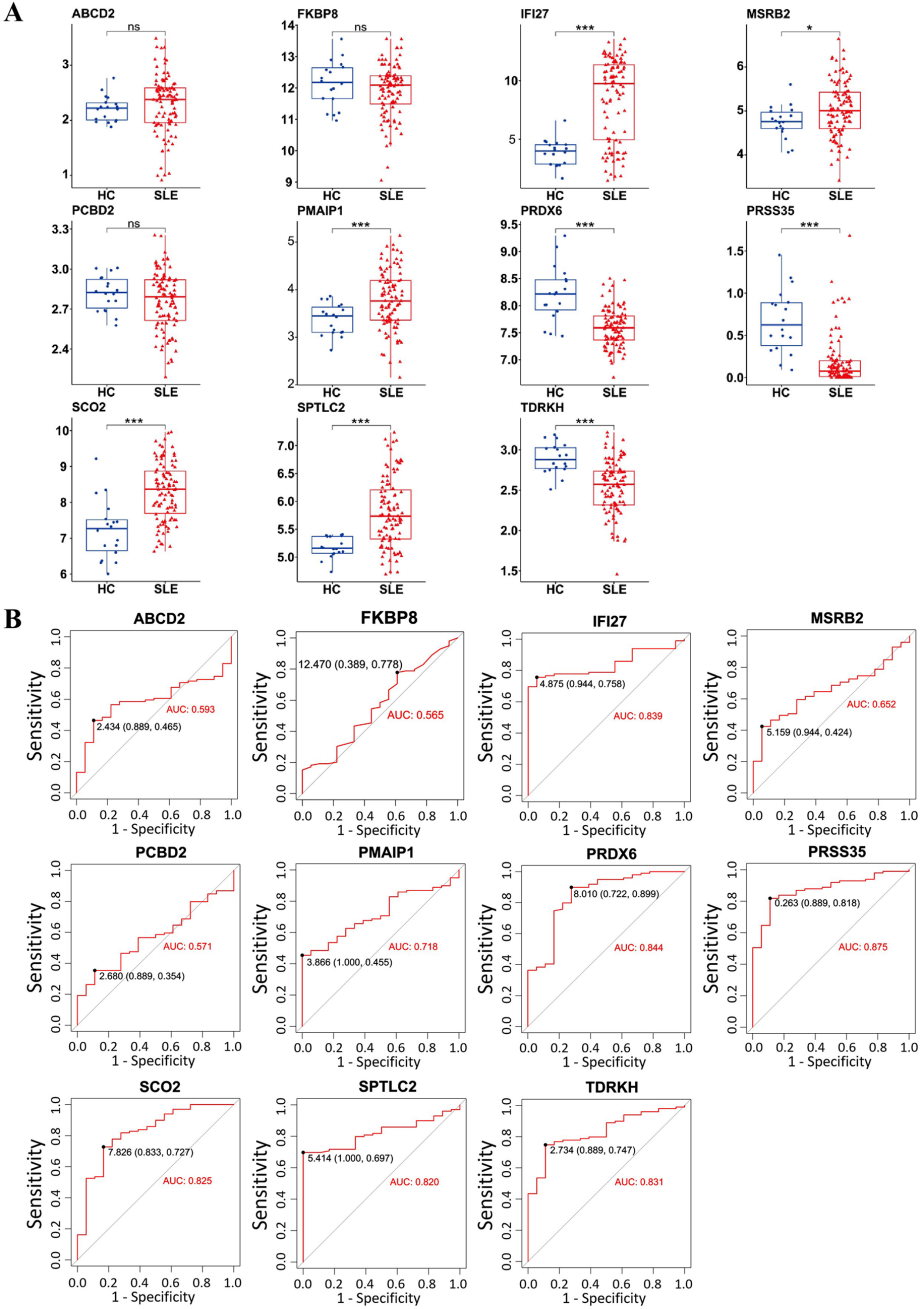
The expression levels of hub genes in the validation datasets. **A** Validation of the expression of diagnostic biomarkers based on the validation datasets (GSE72509). “HC” represented the health control samples, and “SLE” represented the SLE patients. **B** The ROC curve showing the AUC value of IFI27, MSRB2, PMAIP1, SCO2, SPTLC2, ABCD2, FKBP8, PCBD2, PRDX6, PRSS35, and TDRKH based on the data set of the discovery cohort

To evaluate the expression levels of eleven hub genes in SLE, we utilized two datasets (GSE50772 and GSE61635) as the training cohort. Comparative analysis revealed significantly elevated expression levels of five genes (IFI27, MSRB2, PMAIP1, SCO2, and SPTLC2) in SLE patients compared to healthy controls, while six genes (ABCD2, FKBP8, PCBD2, PRDX6, PRSS35, and TDRKH) were downregulated (Fig. 5A). Receiver operating characteristic (ROC) curve analysis demonstrated robust diagnostic performance for all 11 genes in the discovery cohort (Fig. 5B). For validation, we analyzed an independent dataset (GSE72509). Consistent with the training set, the five upregulated genes (IFI27, MSRB2, PMAIP1, SCO2, and SPTLC2) remained significantly elevated in SLE patients (Fig. 6A). However, among the six downregulated genes, only PRDX6 and PRSS35 exhibited consistent suppression (Fig. 6A). ROC analysis further revealed diminished diagnostic efficacy for ABCD2 (AUC = 0.593), FKBP8 (AUC = 0.565), MSRB2 (AUC = 0.652), and PCBD2 (AUC = 0.571) (Fig. 6B), suggesting limited discriminative power for these markers in the validation cohort.

### The validation of the potential diagnostic markers using Chinese cohort revealed that PRDX6 and IFI27 were more likely to be SLE biomarkers for the Chinese population

**Fig. 7:**
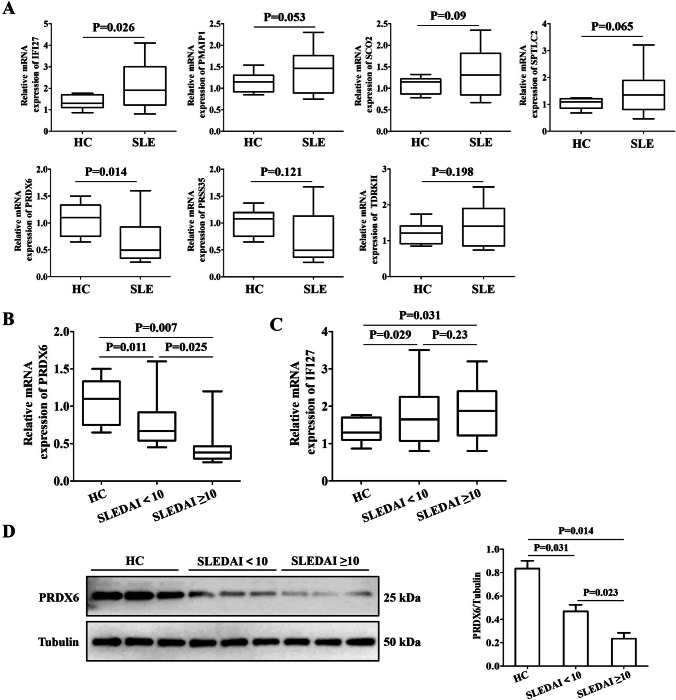
The validation of the putative diagnostic markers by RT-qPCR in the Chinese population using our own cohort. **A** The relative mRNA levels of IFI27, MSRB2, PMAIP1, SCO2, SPTLC2, PRDX6, PRSS35, and TDRKH in the PBMC of SLE patients and healthy control samples. **B** The relative mRNA levels of IFI27 in HC, SLEDAI < 10, and SLEDAI ≥ 10. **C** The relative mRNA levels of PRDX6 in HC, SLEDAI < 10, and SLEDAI ≥ 10. **D** The relative protein levels of Prdx6

SLE is highly heterogeneous among different races and regions. To ensure that the biomarkers we screened could be applied in the Chinese population, we further performed the RT-qPCR assays on the expression of these seven markers in the PBMCs of 70 Chinese SLE patients and 45 Chinese healthy controls. Transcript level analysis demonstrated significant differential expression in SLE patients versus healthy controls, with PRDX6 showing decreased expression and IFI27 exhibiting increased expression (Fig. 7A). The remaining five hub genes showed no statistically significant differences between SLE patients and healthy controls groups (Fig. 7A). Further analysis revealed that the mRNA level of Prdx6 was significantly lower in patients with SLEDAI ≥ 10 compared to those with SLEDAI < 10 (Fig. 7B), whereas IFI27 showed no statistically significant difference between these two groups (Fig. 7C). The Western blot data also showed that the protein level of PRDX6 was significantly lower in patients with SLEDAI ≥ 10 compared to those with SLEDAI < 10 (Fig. 7D). These data suggest that PRDX6 may serve as a potential biomarker for SLE disease progression.

### The PRDX6 mRNA levels correlate with specific clinical manifestations in SLE

**Fig. 8:**
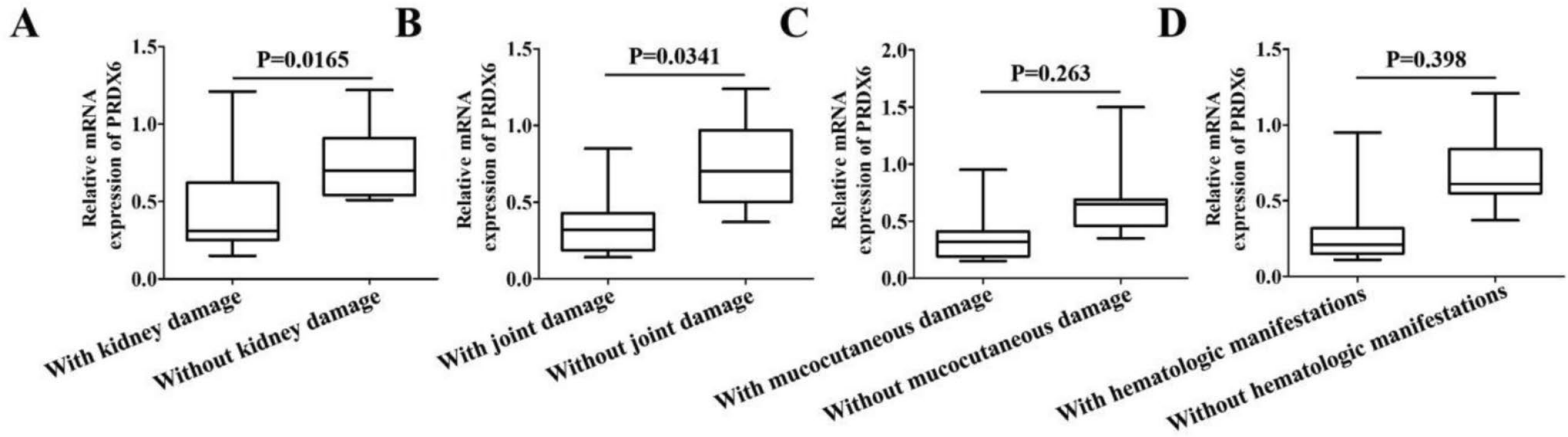
The validation of PRDX6 expression in SLE and health controls. **A** The relative mRNA levels of PRDX6 in renal involvement and without renal involvement of SLE patients. **B** The relative mRNA levels of PRDX6 in joint involvement and without joint involvement of SLE patients. **C** The relative mRNA levels of PRDX6 in cutaneous/mucosal lesions and without cutaneous/mucosal lesions of SLE patients. **D** The relative mRNA levels of PRDX6 in hematologic abnormalities and without hematologic abnormalities of SLE patients

The PRDX6 mRNA levels in PBMCs were significantly lower in SLE patients with renal involvement compared to those without renal involvement (*P* < 0.05) (Fig. 8A) and significantly lower in those with joint involvement than in those without (*P* < 0.01) (Fig. 8B). No statistically significant differences were observed in patients with or without cutaneous/mucosal lesions or hematologic abnormalities (Fig. 8C, D). PRDX6 mRNA expression in SLE patient PBMCs was negatively correlated with SLEDAI scores (*r* = − 0.502, *P* < 0.001) (Table 3). No significant correlations were found between PRDX6 mRNA expression and ESR, CRP, complement C3 or C4, anti-dsDNA antibody levels, WBC, or PLT counts (Table 3).

**Table 3:** Correlation of PBMC PRDX6 mRNA levels with key laboratory parameters in SLE

Laboratory test data	*r*	*P*
ESR (mm/h)	− 0.117	0.336
CRP (mg/L)	− 0.226	0.060
Complement C3 (mg/L)	0.095	0.433
Complement C4 (mg/L)	0.021	0.866
Anti-dsDNA antibody (IU/mL)	− 0.004	0.971
SLEDAI score	− 0.502	< 0.001
WBC (× 10^9^/L)	− 0.064	0.601
PLT (× 10^9^/L)	− 0.024	0.884

### The ratio changes of immune cells in SLE patients and their correlation with the expression of IFI27 and PRDX6

**Fig. 9:**
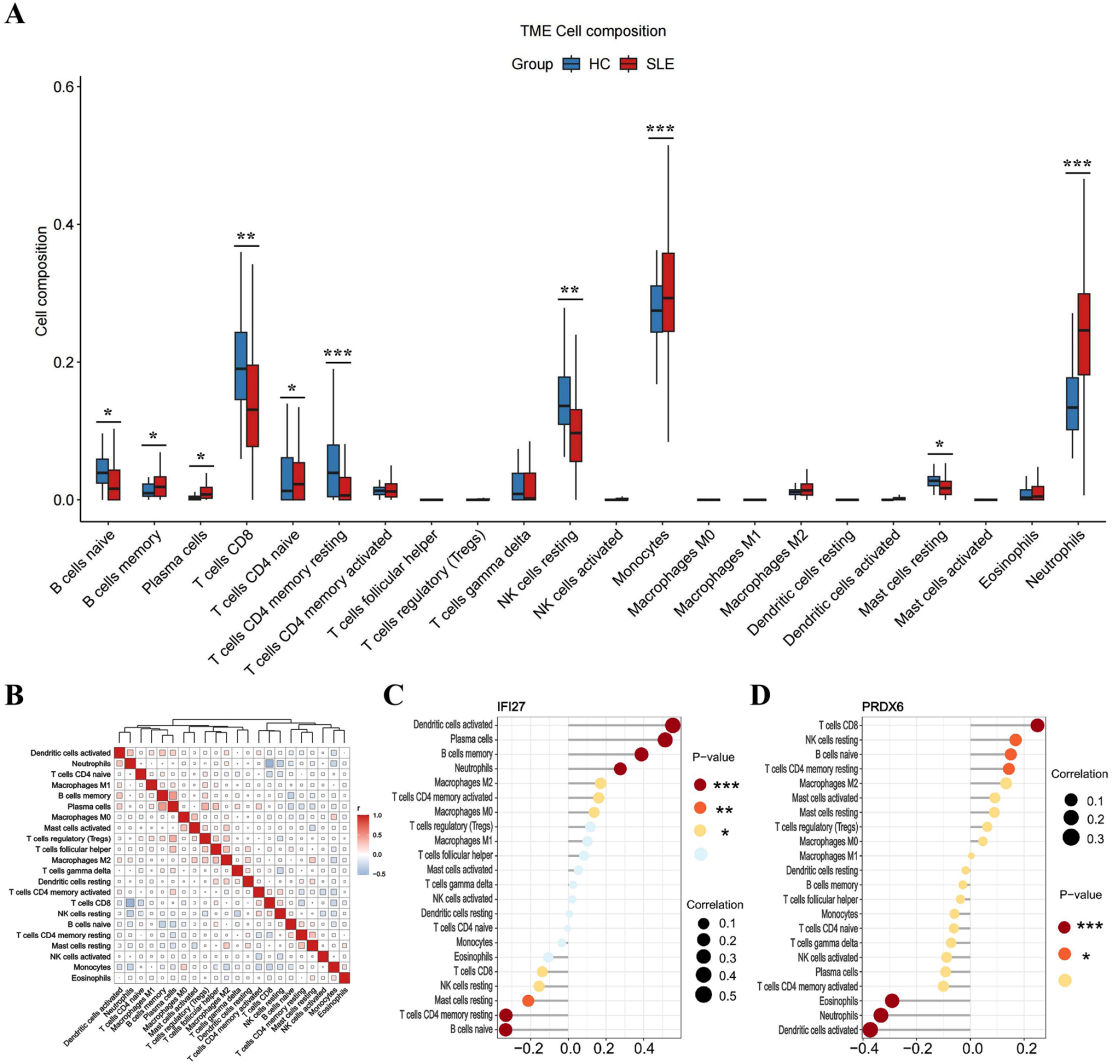
The ratios of immune cells in the PBMC of SLE patients and their correlations with the biomarkers’ expression. **A** Violin diagram showing the proportion of 22 types of immune cells in the PBMC of SLE patients and healthy people. **B** The heatmap showing the proportion correlation between the 22 types of immune cells in SLE patients. The size of the colored squares represented the strength of the correlation, and red represented a positive correlation, and blue represented a negative correlation. The correlation between **C** IFI27 and **D** PRDX6 expression with the degrees of immune cells in SLE. The size of the dots represented the strength of the correlation. The color of the dots represented the *P* value. *P* < 0.05 was considered statistically significant

The onset of SLE causes changes in the proportion and function of a series of immune cells. To find the biomarkers whose expression was correlated with the proportions of immune cells, we first analyzed the ratio changes of 22 immune cells in 160 SLE patients and 50 healthy people using the CIBERSORT algorithm. The results showed that compared with the control group, the proportions of naive B cells, CD8 T cells, CD4 memory resting T cells, resting natural killer (NK) cells, and resting mast cells in SLE were significantly lower, while memory B cells, plasma cells, naive CD4 T cells, monocytes, and neutrophils were higher (Fig. 9A).

Following that, we investigated the correlation between the ratios of the 22 types of immune cells in SLE patients and discovered that the degrees of memory B cells and plasma cells, the levels of plasma cells and regulatory T cells (Tregs), all had a strong positive link, respectively. Furthermore, the ratio of neutrophils was adversely linked with that of CD8 T cells, resting NK cells, and monocytes (Fig. 9B). We further analyzed the correlation between the ratios of the 22 types of immune cells and the expression of PRDX6 and IFI27 in SLE patients. The IFI27 expression was positively correlated with the ratios of activated dendritic cells, plasma cells, memory B cells, and neutrophils and negatively correlated with activated naive B cells and resting memory CD4 T cells (Fig. 9C). The PRDX6 expression was positively correlated with the ratios of CD8 T cells, resting NK cells, naive B cells, and resting CD4 memory T cells and negatively correlated with activated dendritic cells, neutrophils, and eosinophils (Fig. 9D).

## Discussion

SLE is a multi-system autoimmune disease driven by genetic susceptibility and environmental triggers, which collectively disrupt immune homeostasis. Pathogenic T/B-cell dysregulation and immune complex deposition trigger systemic inflammation, resulting in diverse clinical presentations [9]. The disease heterogeneity and lack of specific biomarkers underscore the urgent need for novel diagnostic and therapeutic targets.

Previous study has revealed several potential SLE biomarkers of mitochondria-related genes based on three machine learning methods: RF, SVM, and LASSO [10]. They did not evaluate the diagnostic performance of the identified mitochondrial-related hub genes using ROC curve analysis. Our analysis indicates that MSRB2, one of their proposed hub genes, exhibits substantially limited diagnostic utility (AUC = 0.652). They also failed to validate the potential hub genes in the Chinese cohort. In the present study, we further validated the screened markers using our own Chinese cohort; thereby, the markers had potential application for Chinese patients. In the SLE candidate markers, PRDX6 is identified for the first time. Furthermore, we confirmed the expression of PRDX6 in the Chinese population using our own cohort.

Our analysis revealed associations between specific immune cell infiltration patterns and biomarker gene expression. However, these findings require further validation across diverse clinical contexts, and the biological significance of these correlations remains unclear. Notably, the observed relationships may reflect indirect effects rather than direct causation. For instance, systemic inflammation in lupus could concurrently alter immune cell distributions and modulate marker gene expression.

Our integrated bioinformatics analysis of two independent datasets identified 11 mitochondrial-related hub genes potentially implicated in SLE pathogenesis and treatment. Mitochondria serve as central regulators of cellular metabolism and immune function. Their dysfunction triggers innate immune activation and promotes inflammatory/autoimmune responses [11]. In SLE pathogenesis, mitochondrial abnormalities impair immune cell metabolism and function, while inducing pro-inflammatory mediator release that exacerbates disease progression [12, 13]. PRDX6 is a key antioxidant enzyme maintaining redox homeostasis and cellular oxidative stress responses [14]. Regarding PRDX6 specifically, a study in Korean SLE patients found significantly higher urinary PRDX6 levels in both SLE and lupus nephritis patients compared to controls, with levels positively correlating with anti-double-stranded DNA antibody [15]. A recent study in a Chinese cohort also identified a strong genetic association between the PRDX6 antisense RNA (PRDX6-AS1) and SLE susceptibility, reporting upregulated PRDX6-AS1 expression in SLE patients [16]. Notably, PRDX6 was significantly downregulated in SLE patients versus healthy controls. This finding aligns with established evidence of systemic oxidative/anti-oxidative imbalance in SLE [17], including reports of elevated oxidative stress independent of renal involvement [18]. The observed PRDX6 deficiency suggests its potential pathogenic role in SLE, possibly through impaired oxidative stress regulation. Experimental validation corroborated the bioinformatics findings, demonstrating significantly reduced PRDX6 mRNA and protein expression in the PBMCs of SLE patients compared to healthy controls. Importantly, PRDX6 levels showed progressive downregulation with worsening disease activity (moderate-severe vs mild/inactive SLE) and exhibited a strong inverse correlation with SLEDAI scores. Clinical stratification revealed particularly pronounced PRDX6 suppression in patients with renal or joint involvement, while levels remained comparable in those with cutaneous/mucosal or hematologic manifestations. This organ-specific expression pattern positions PRDX6 as a promising biomarker for predicting lupus nephritis and arthritis progression.

With respect to articular involvement, previous studies have demonstrated decreased serum PRDX6 levels in rheumatoid arthritis (RA) patients through ELISA assay [19]. PRDX6 may drive the pathogenesis of SLE associated joint damage through its canonical role in suppressing NF-κB transcriptional activation. Diminished PRDX6 expression may lead to enhanced NF-κB activation, subsequent elevation of pro-inflammatory cytokines (IL-1β and IL-18), and promotion of Th17 cell polarization [20]. Notably, as the most frequently affected organ in SLE, renal injury involves multi-factorial mechanisms including immune complex deposition, inflammatory cell infiltration, and oxidative stress [21]. The observed downregulation of PRDX6 mRNA in PBMCs from SLE patients with renal involvement may reflect its established role in mitigating oxidative stress through the Nrf2-mediated antioxidant pathway [22]. Currently, the association between PRDX6 expression and systemic organ damage (particularly articular and renal involvement) in SLE remains unexplored, and the precise mechanisms underlying these associations remain to be elucidated in future research. Interestingly, PRDX6 expression trends (inverse with ESR/CRP/anti-dsDNA; positive with C3/C4) may indicate biological links obscured by sample limitations or antioxidant redundancy, requiring expanded cohorts for verification.

## Conclusions

This study demonstrated that PRDX6 is significantly downregulated in SLE patients at both mRNA and protein levels, supporting its potential role as a driver of oxidative stress and immune imbalance in SLE. Restoring redox homeostasis via PRDX6 may offer a novel therapeutic avenue, but its mechanistic role in SLE pathogenesis remains to be fully characterized.

## Data Availability

All gene expression data used in this study are publicly available from the Gene Expression Omnibus (GEO) under accession numbers GSE50772 and GSE61635 (https://www.ncbi.nlm.nih.gov/geo/). The mitochondrial gene list was retrieved from the Mitocarta3.0 database (https://www.broadinstitute.org/mitocarta).
